# Hydrothermal Separation of Titanium Vanadium and Chromium from a Pregnant Oxalic Acid Leachate

**DOI:** 10.3390/ma15041538

**Published:** 2022-02-18

**Authors:** Zihui Dong, Jie Zhang, Baijun Yan

**Affiliations:** State Key Laboratory of Advanced Metallurgy, Department of Physical Chemistry of Metallurgy, School of Metallurgical and Ecological Engineering, University of Science and Technology Beijing, Beijing 100083, China; dongzihui2017@126.com (Z.D.); zhangj@ustb.edu.cn (J.Z.)

**Keywords:** vanadium slag, hydrothermal method, extraction, stripping

## Abstract

The separation of titanium, vanadium and chromium in vanadium slag (VS) is a difficult problem restricting the comprehensive utilization of VS. This paper presents the first study on the separation of titanium, vanadium and chromium from oxalic acid leachate of VS. Firstly, the separation of titanium from the leachate by hydrothermal method was studied. The results show that more than 99% of titanium in the leachate was precipitated in the form of spherical anatase TiO_2_ with the purity of 95.7%. Then, the extraction separation of vanadium and chromium from the titanium-free filtrate by three-stage extraction of acidified N235 extractant and four-stage stripping of HCl solution was investigated. The extraction mechanism was identified as the anion exchange reaction between acidified N235 extractant and vanadium and chromium complex anions, which were further stripped by HCl solution in the stripping process. After obtaining the concentrated and purified stripping solution containing vanadium and chromium, the separation of vanadium and chromium from the stripping solution by hydrothermal method was studied, and the product was mainly composed of VO_2_ and Cr_2_O_3_. This process provides an idea for the comprehensive utilization of titanium, vanadium and chromium in oxalic acid system.

## 1. Introduction

Vanadium, titanium and chromium are significant strategic metals which are widely used in aerospace, energy and alloy fields, etc. [1]. Vanadium slag (VS) is a by-product of smelting vanadium titanomagnetite, which is mainly composed of vanadium, titanium, chromium, iron, manganese and silicon [2,3]. However, as a high-quality raw material containing vanadium, titanium and chromium, only the vanadium bearing in VS can be extracted through traditional processes, namely a sodium salt roasting–water leaching process and calcification roasting–acid leaching process [4,5]. As for the principle of these processes, it can be summarized as follows. During the roasting step, vanadium (III) occurred in VS is oxidized and reacts with sodium salt or calcium salt to form soluble vanadate. Then, vanadium pentoxide (V_2_O_5_) is produced by leaching, purification, precipitation and calcination processes [6]. Unfortunately, the valuable titanium and chromium are not extracted and left in tailings, resulting in a waste of resources and environmental pollution [7]. In view of the above problems, the sub-molten salt method with the characteristics of co-extraction of vanadium and chromium was proposed. Insoluble vanadium (III) and chromium (III) in VS are oxidized and leached in a high-concentration sodium hydroxide medium under oxygen pressure, but the titanium is still not extracted [8]. To solve the above problems, in our previous research [9], a new method for co-extracting vanadium, titanium and chromium from vanadium slag by oxalic acid hydrothermal leaching was proposed, by which the vanadium, titanium and chromium were extracted from VS directly and simultaneously under hydrothermal conditions by oxalic acid solution. The leaching extents of vanadium, titanium and chromium can reach 97.9, 98.4 and 93.3%. However, confronting this pregnant oxalic acid leachate, how to separate the vanadium, titanium and chromium becomes an urgent and important problem due to scarce attention paid to this complex organic acid solution system and thus, few studies carried out on it.

Based on our previous study and other published results [10,11,12], it can be found that under appropriate hydrothermal conditions, the vanadium complex ions that occurred in oxalic acid solution began to decompose and precipitate as vanadium oxide. Thus, an attempt of sequential hydrothermal separation of vanadium, titanium and chromium from the pregnant oxalic acid leachate was made in the present study. Fortunately, the results show that titanium oxide precipitates firstly from the pregnant leachate at a relatively low hydrothermal temperature, while the vanadium and chromium complex ions can remain in the solution. After filtering out the titanium oxide, a titanium-free filtrate (TFF) containing vanadium, chromium and impurities was obtained. Then, the next problem is how to separate vanadium and chromium from the TFF.

Considering the TFF after titanium separation, it is characterized by relatively low concentrations of vanadium and chromium and high contents of impurities. Therefore, it is better to purify the TFF and upgrade the concentrations of vanadium and chromium before further treatment. As for the purification and enrichment, solvent extraction and stripping are commonly adopted operations. Some investigations about the extraction of vanadium in oxalic acid leachate have been reported. For example, it was reported that about 99% of vanadium in oxalic acid leachate of waste catalyst can be extracted with Alamine-336 extractant by two-stage extraction and about 99.64% of vanadium in the oxalic acid leachate of shale can be extracted with Aliquat-336 extractant by five-stage extraction. The extracting mechanism was assumed to be the replacement of Cl^−^ ion in Aliquat-336 extractant by VO(C_2_O_4_)_2_^2−^ complex anion in leachate [13,14]. Comparably, the vanadium (III and IV) in the present obtained TFF was identified to occur as V(C_2_O_4_)_3_^3−^ and VO(C_2_O_4_)_2_^2−^, and chromium (III) mainly exists in the form of Cr(C_2_O_4_)_3_^3−^. Therefore, an acidified anionic N235 extractant was selected as the co-extractant of vanadium and chromium from the filtrate.

Based on the above analysis, a process for separating titanium, vanadium and chromium from the oxalic acid leachate of VS was proposed and investigated systematically in the present study. In summary, this new process consists of the following steps: firstly, the titanium is precipitated from the oxalic acid leachate by hydrothermal method; next, the vanadium and chromium left in the TFF are extracted by acidified N235 extractant and separated from the impurities; then, the vanadium and chromium are stripped by HCl solution; finally, the vanadium and chromium in the stripping solution are coprecipitated by a hydrothermal method.

## 2. Materials and Methods

### 2.1. Materials

The titanium-, vanadium- and chromium-bearing oxalic acid leachate of VS was prepared by hydrothermal leaching, and the detailed preparation procedure can be found in our previous publication [9]. For brevity, only the main leaching parameters are provided here. The VS from HBIS Group ChengSteel of Chengde, China was hydrothermally leached at 125 °C for 90 min, with oxalic acid concentration of 25 wt% (Beijing Honghu United Chemical Products Co., Ltd., Beijing, China), liquid–solid mass ratio of 8:1 and iron powder of 3.2 wt% (Sinopharm chemical reagent Beijing Co., Ltd., Beijing, China). The components of the leachate analyzed by ICP Optical Emission Spectrometer (ICP-OES, OPTIMA 7000DV, Waltham, MA, USA) are listed in Table 1. It can be seen that, in addition to the principal component V, Ti and Cr, a certain amount of impurities of Fe, Mn, Si, Ca, Mg and Al exist in the leachate. The initial pH of the leachate was measured to be about 0.7.

In addition, the N235 extractant (AR) and 2-Octanol phase modifier (AR) produced by Beijing Honghu United Chemical Products Co., Ltd., Beijing, China and the kerosene diluent (AR) produced by Beijing Tongguang Fine Chemical Co., Ltd., Beijing, China. were used.

### 2.2. Procedure

**Figure 1:**
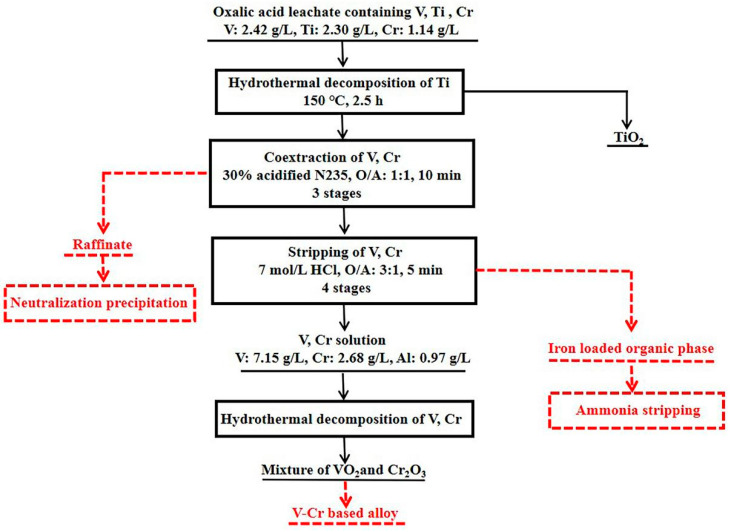
Flow diagram of separating titanium, vanadium and chromium from the oxalic acid leachate of vanadium slag.

The flow diagram of separating titanium, vanadium and chromium from the oxalic acid leachate of VS was proposed, as shown in Figure 1, including the following three segments. The first is the hydrothermal precipitation of titanium from the leachate to obtain TiO_2_ directly. The second is the extraction separation of vanadium and chromium from the TFF by solvent extraction, and the stripping solution enriching vanadium and chromium was obtained. The third is the hydrothermal precipitation of vanadium and chromium from the stripping solution. The product was mainly composed of VO_2_ and Cr_2_O_3_, which can be used to produce V-Cr based alloy.

#### 2.2.1. Hydrothermal Precipitation of Titanium

The first step is to separate the component titanium from the oxalic acid leachate by hydrothermal precipitation in a 50 mL autoclave (Jieang Instrument Co., Ltd., Shanghai, China), and the influences of reaction temperature and reaction time on the precipitation process were investigated. Specifically, 15 mL leachate was loaded into an autoclave, and the experiment was performed after setting the reaction temperature and reaction time. After the experiment, the autoclave was cooled to room temperature. Then, the solid and liquid were separated by filtration and washing. The solid product was dried in an oven at 60 °C for 5 h for X-ray diffraction analysis (XRD, MAC Science Co. Ltd., Kanagawa, Japan), Field Emission SEM analysis (SEM, JSM-6701F, Beijing, China), Fourier transform infrared spectroscopy analysis (FTIR, Thermo Fisher Nicolet iS50 spectrometer, Shanghai, China), and the liquid was analyzed by ICP Optical Emission Spectrometer (ICP-OES, OPTIMA 7000DV, Waltham, MA, USA) to determine the concentrations of ions. The precipitation extent (*η*) was calculated by Equation (1).

(1) $$\eta =(1-\frac{C_{f}V_{f}}{C_{r}V_{r}})\times 100\%$$

where, *η* is the precipitation extent (%); *C_r_* and *C_f_* represent the ion concentration in liquid before and after hydrothermal experiment (g/L), respectively; *V_r_* and *V_f_* represent the volume of liquid before and after hydrothermal experiment (L), respectively. 

#### 2.2.2. Extraction Separation of Vanadium and Chromium

After the component titanium was hydrothermally precipitated from the oxalic acid leachate, the components vanadium and chromium left in TFF were separated by the solvent extraction method. The organic solvent N235 (Beijing Honghu United Chemical Products Co., Ltd., Beijing, China) acidified with HCl solution was used as the extractant, and the acidification was performed by stirring the mixture of N235 and equal volume 1 mol/L HCl solution with stirring speed of 600 r/min for 30 min. Then, the extractant was mixed with the filtrate and stirred in a beaker at the speed of 500 r/min. Thereafter, the loaded organic phase (LOP) and the raffinate were separated in a separation funnel. Finally, the concentrations of ions in the raffinate were measured by ICP, and the extraction extent (*E*) was calculated by Equation (2).

(2) $$E=(1-\frac{C_{1}}{C_{0}})\times 100\%$$

where, *E* is the extraction extent (%); *C*_0_ is the ion concentration in extract (g/L); *C*_1_ is the ion concentration in raffinate (g/L).

To further recover the components vanadium and chromium from the loaded organic phase, a stripping step was performed. The LOP was mixed with stripping agent HCl in a beaker and stirred with a speed of 500 r/min. After the reaction, the lean organic phase and the stripping solution were separated in a separation funnel. The stripping extent (*S*) was calculated by Equation (3).

(3) $$S=(\frac{V_{A_{q.}}^{\prime }\;C_{A_{q.}}^{\prime }}{V_{Org.}{\;C}_{Org.}})\times 100\%$$

where, *S* is the stripping extent (%); *V′_A_*_*q*._ and *V_Org._* represent the volumes of stripping solution and LOP (L), respectively; *C′_A_*_*q*._ and *C_Org._* represent the ion concentration in stripping solution and in organic phase (g/L), respectively.

#### 2.2.3. Hydrothermal Precipitation of Vanadium and Chromium from Stripping Solution

Firstly, the pH value of stripping solution was adjusted to about 0.7 using sodium hydroxide. Then, 20 mL stripping solution was loaded into a 50 mL autoclave, and the experiment was performed after setting the reaction temperature and reaction time. After solid–liquid separation, the concentrations of ions in liquid and the phase of the product were analyzed by ICP and XRD. The *η* was calculated by Equation (1).

## 3. Results and Discussion

### 3.1. Hydrothermal Precipitation of Titanium from the Pregnant Leachate

#### 3.1.1. Influence of Reaction Temperature on Titanium Precipitation

The reaction temperature is an important factor in hydrothermal experiments. Therefore, the influence of reaction temperature on the *η*s was studied preferentially. The experiments were carried out at the reaction temperature of 140–170 °C and the reaction time of 1.5 h.

**Figure 2:**
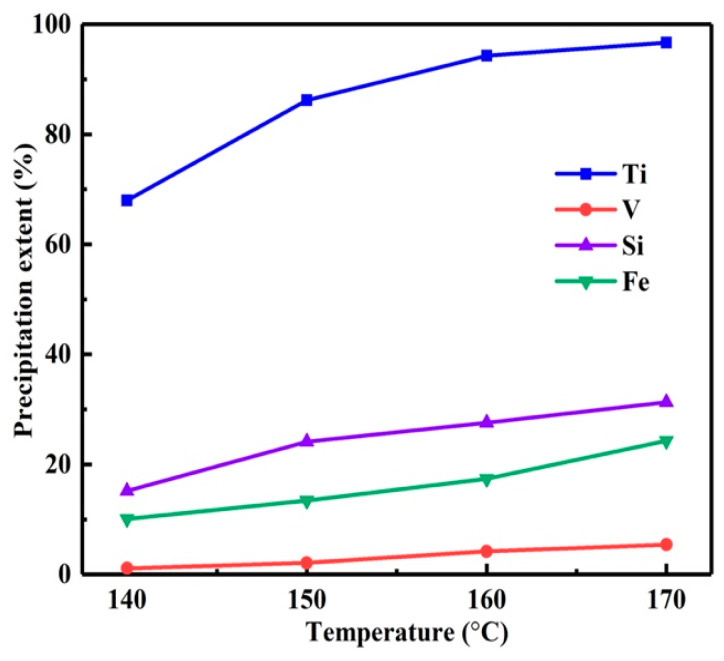
Influence of reaction temperature on precipitation extents.

As shown in Figure 2, with the temperature varied from 140 to 170 °C, the *η* of titanium increased from 68.1 to 96.7%. However, when the temperature exceeded 150 °C, the *η*s of impurities also increased significantly. The *η* was calculated by Equation (1). This led to the low purity of titanium dioxide and the loss of high-value vanadium. Therefore, the preferred reaction temperature was 150 °C. 

**Figure 3:**
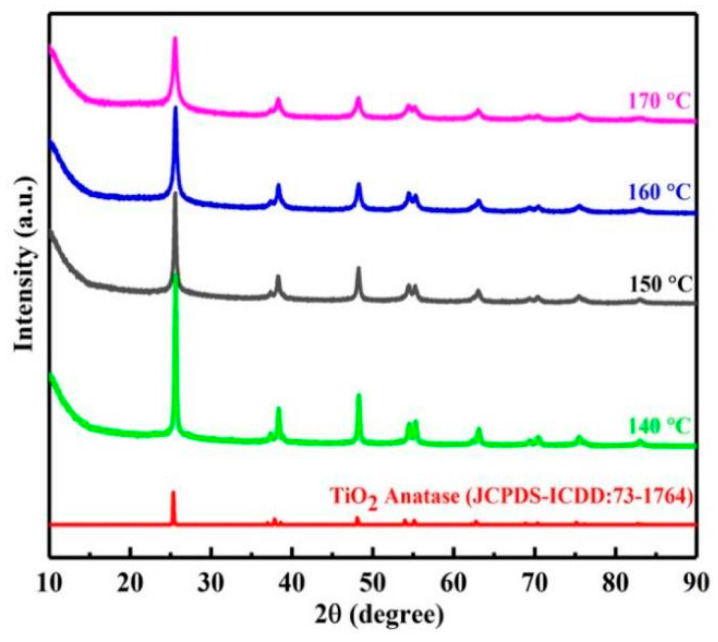
XRD of products at different temperatures.

In order to clarify the hydrothermal decomposition process of leachate, the phase transition of products at different temperatures was characterized by XRD. As shown in Figure 3, the hydrothermal decomposition product of titanium was anatase titanium dioxide. However, with the increase in temperature, the characteristic peak intensity of titanium dioxide became weaker and the half peak width became wider. This may be caused by the production of amorphous silica. Therefore, in order to further understand the changes of impurities in hydrothermal decomposition process, the FTIR spectra of products at different temperatures were studied.

**Figure 4:**
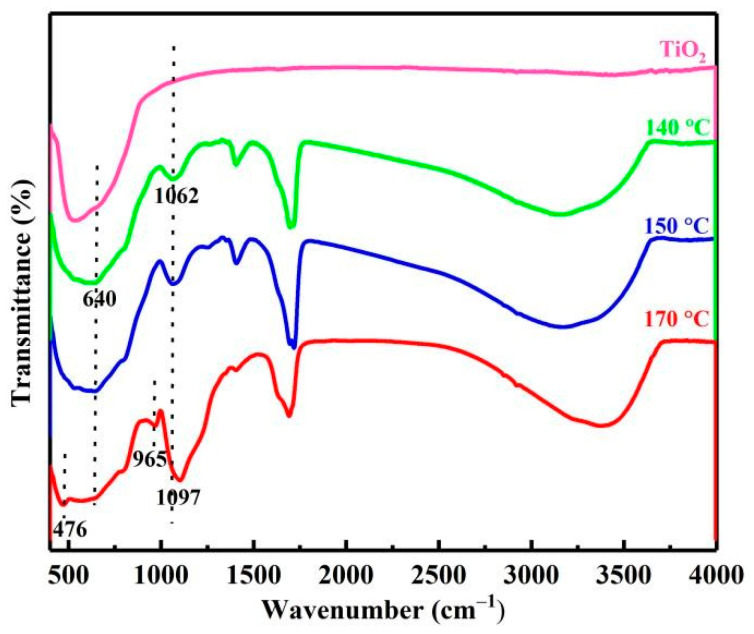
FTIR of products at different temperatures.

As can be seen from Figure 4, compared with the FTIR spectra of pure titanium dioxide, the characteristic peaks of impurities in the product were detected. Specifically, for impurity silicon, the peak at 1000–1250 cm^−1^ with large strength and a wide shape was attributed to the characteristic peak of Si-O-Si asymmetric stretching vibration, and the peak at 965 cm^−1^ belonged to the characteristic peak of Si-OH stretching vibration, indicating the formation of amorphous silicon dioxide. As the temperature rose from 140 °C to 170 °C, the intensity of the characteristic peak at 1000–1250 cm^−1^ increased, and 1062 cm^−1^ at the low wavenumber was shifted to 1097 cm^−1^ at the high wavenumber due to the increase in amorphous silica content [15].

For the impurity vanadium, the characteristic peaks of vanadium oxide should be concentrated in 400–1000 cm^−1^, most of which may be overlapped by the peaks of titanium dioxide. However, the V=O characteristic peak of vanadium dioxide (VO_2_) at 640 cm^−1^ was detected [16], which proved that vanadium dioxide (VO_2_) was a decomposition product of vanadium. Similarly, for impurity iron, the Fe-O characteristic peak of α-Fe_2_O_3_ at 476 cm^−1^ was detected at 170 °C, indicating that iron will precipitate in the form of Fe_2_O_3_ with the increase in temperature [17].

#### 3.1.2. Influence of Reaction Time on Titanium Precipitation

**Figure 5:**
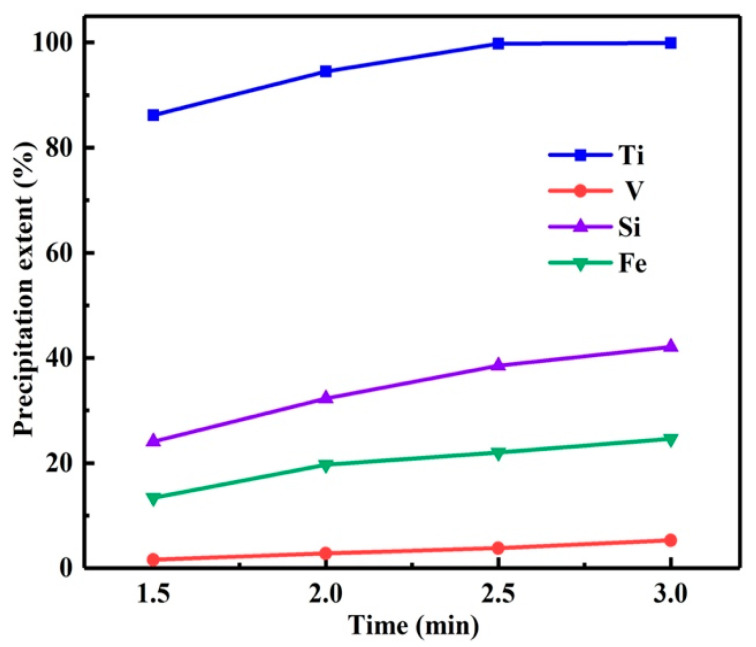
Influence of reaction time on precipitation extents.

Reaction time is another important factor in hydrothermal experiments, which affects the *η* and the micromorphology of the product. Therefore, the influence of reaction time on the *η* was investigated with the reaction temperature of 150 °C and the reaction time of 1.5 h–3 h. As depicted in Figure 5, prolonging the reaction time can improve the *η* of titanium, but it will also lead to the coprecipitation of impurities such as silicon, iron and vanadium.

**Figure 6:**
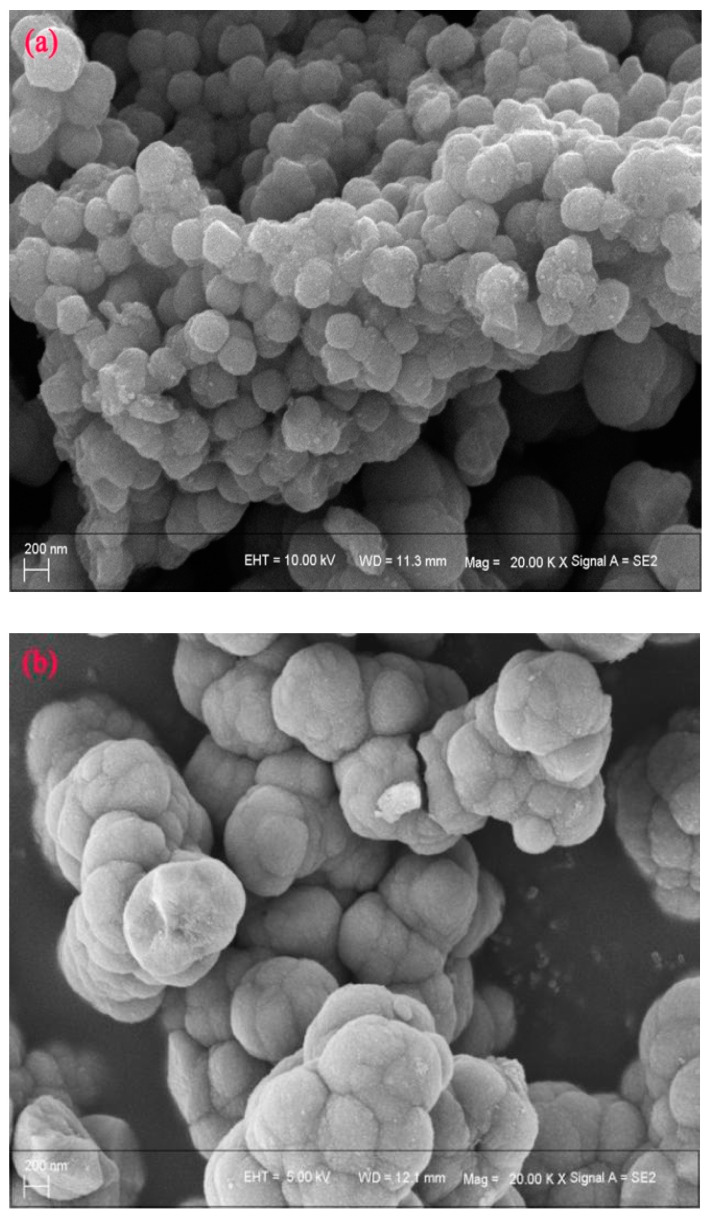
The micromorphology of products at 1.5 h (**a**) and 2.5 h (**b**).

In order to further clarify the micromorphology change of titanium dioxide during hydrothermal decomposition, SEM analysis was performed. As shown in Figure 6, when the reaction time was 1.5 h, the particle size of titanium dioxide was uniformly about 200 nm, while when the reaction time was 2.5 h, the titanium dioxide particles aggregated and grew about 600 nm. Therefore, considering the *η* of titanium and the morphology of titanium dioxide, the reaction time was selected as 2.5 h.

#### 3.1.3. Characteristics of the Precipitated Titanium Oxide

**Figure 7:**
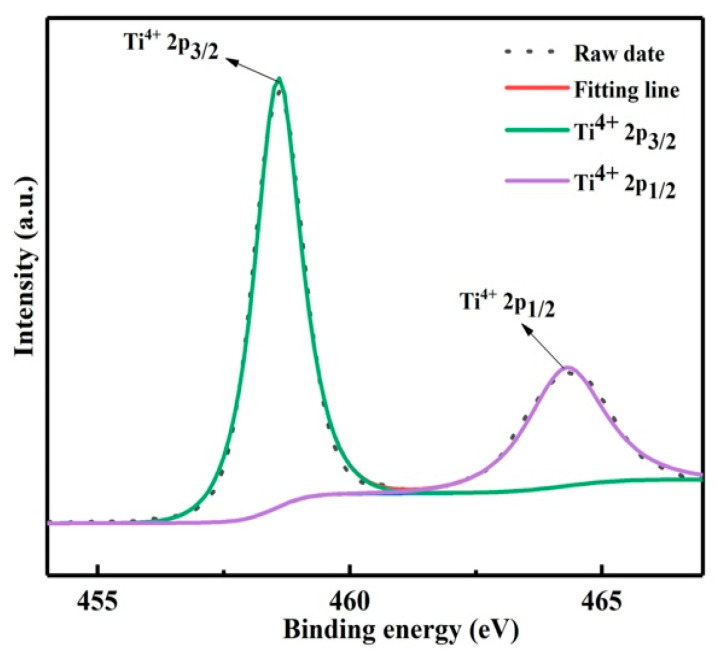
XPS survey spectrum of Ti 2p.

The decomposition product was prepared at 150 °C for 2.5 h. The valence state of titanium in the product was characterized by XPS, as shown in Figure 7, the binding energy peaks at 458.3 and 464.3 eV were assigned to Ti^4+^ 2p_3/2_ and Ti^4+^ 2p_1/2_ [18]. Therefore, titanium in the product exists in the form of anatase titanium dioxide with +4 valence. In addition, the purity of the product was analyzed by ICP, as shown in Table 2; the purity of titanium dioxide was 95.7%. 

### 3.2. Solvent Extraction of Vanadium and Chromium from the TFF

#### 3.2.1. Mechanism Analysis of Extraction Process

**Figure 8:**
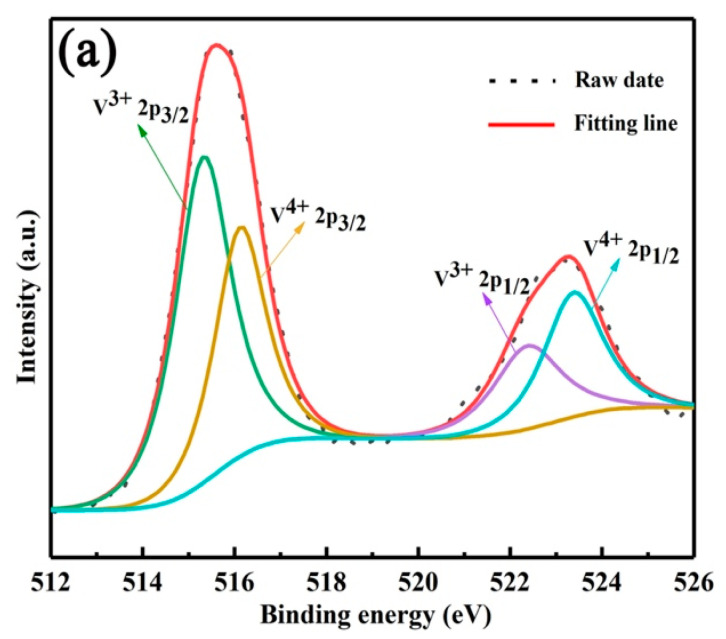

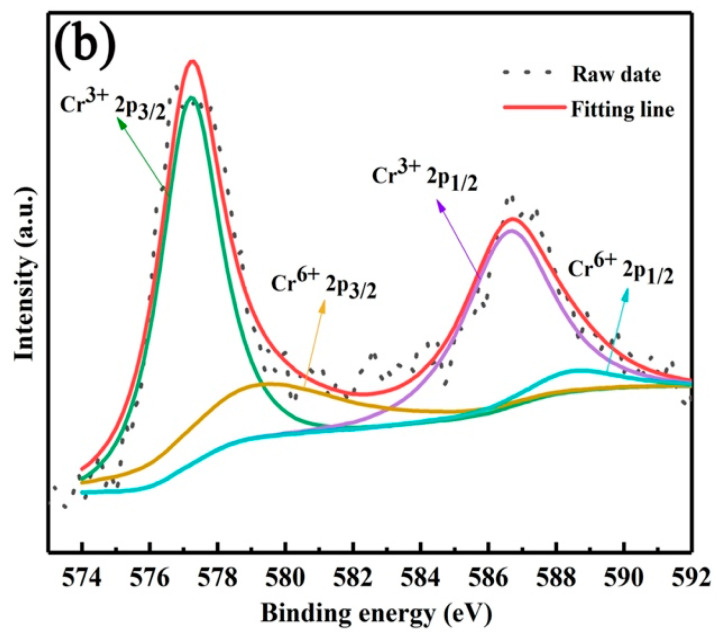
XPS survey spectrum of V 2p (**a**) and Cr 2p (**b**).

In order to clarify the extraction mechanism, the occurrence states of vanadium, chromium, iron and aluminum ions in the extract were studied. Firstly, the valence states of vanadium and chromium ions were analyzed by XPS, as shown in Figure 8a, the binding energy peaks at 515.29 and 522.61 eV were attributed to V^3+^ 2p_3/2_ and V^3+^ 2p_1/2_, and the binding energy peaks at 516.2 and 523.4 eV were assigned to V^4+^ 2p_3/2_ and V^4+^ 2p_1/2_. Therefore, the vanadium ions in the extract are composed of V^3+^ and V^4+^ [19]. The XPS results of chromium ions are shown in Figure 8b, the binding energies of 577.1 and 586.5 eV belonged to 2p_3/2_ and 2p_1/2_ of Cr^3+^, and the binding energies of 579.2 and 588.5 eV belonged to 2p_3/2_ and 2p_3/2_ of Cr^6+^. Therefore, chromium ions in the extract mainly exist in the form of Cr^3+^ and a small amount of Cr^6+^ [18,20].

**Figure 9:**
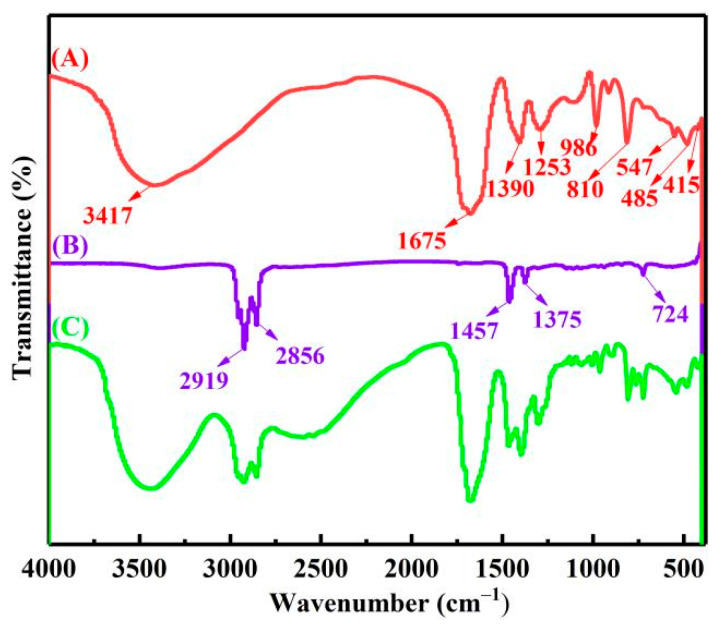
The FTIR of extract (A) 30% N235 + 10% 2−octanol + 60% kerosene organic phase (B) loaded organic phase (C).

Then, the complexing states of vanadium, chromium, iron and aluminum ions in the extract were analyzed by FTIR. As shown in Figure 9A the peak at 3417 cm^−1^ belonged to the O−H stretching vibrations of crystal water [21]. The peak at 1675 cm^−1^ was identified as the asymmetrical deformation vibrations ν_as_(C=O). The peak at 1390 cm^−1^ was assigned to the symmetrical deformation vibrations ν_s_(C−O) + ν(C−C) stretching band. The peak at 1253 cm^−1^ was assigned to the symmetrical deformation vibrations ν_s_(C−O) + δ(O−C=O) bending vibration. The peak at about 810 cm^−1^ was attributed to the bending vibration δ(O−C=O) + ν(M−O) stretching band, M represent elements V, Cr, Fe, Al. The peaks at 986 and 547 cm^−1^ belonged to the V=O and V−O. The peak at about 485 cm^−1^ was attributed to the stretching vibration ν(Al−O) and ν(Fe−O). The peak at about 415 cm^−1^ was attributed to the stretching vibration ν(Cr−O). [22,23,24]. Therefore, complex ions of vanadium, chromium, iron and aluminum in the extract should exist in the form of V(C_2_O_4_)_3_^3−^, VO(C_2_O_4_)_2_^2−^, Cr(C_2_O_4_)_3_^3−^, Fe(C_2_O_4_)_3_^3−^ and Al(C_2_O_4_)_3_^3−^.

Figure 9B shows the FTIR spectra of the organic phase, with characteristic peaks of 2919, 2856, 1457, 1375 and 724 cm^−1^. The FTIR spectra of the LOP is shown in Figure 9C. After extraction, the complex anions of vanadium, chromium, iron and aluminum in the extract were anion exchanged with Cl^−^ in the acidified N235 extractant, and transferred to the organic phase. Therefore, the extraction process can be represented by Equation (4)–(9).

(R_3_N)_org_ + HCl =(R_3_NHCl)_org_ (4)

3(R_3_NHCl)_org_ + V(C_2_O_4_)_3_^3−^ = ((R_3_NH)_3_·V(C_2_O_4_)_3_)_org_ + 3Cl^−^ (5)

2(R_3_NHCl)_org_ + VO(C_2_O_4_)_2_^2−^ = ((R_3_NH)_2_·VO(C_2_O_4_)_2_)_org_ + 2Cl^−^ (6)

3(R_3_NHCl)_org_ + Cr(C_2_O_4_)_3_^3−^ = ((R_3_NH)_3_·Cr(C_2_O_4_)_3_)_org_ + 3Cl^−^ (7)

3(R_3_NHCl)_org_ + Fe(C_2_O_4_)_3_^3−^ = ((R_3_NH)_3_·Fe(C_2_O_4_)_3_)_org_ + 3Cl^−^ (8)

3(R_3_NHCl)_org_ + Al(C_2_O_4_)_3_^3−^ = ((R_3_NH)_3_·Al(C_2_O_4_)_3_)_org_ + 3Cl^−^ (9)

#### 3.2.2. Influence Factors of Extracting Process

**Figure 10:**
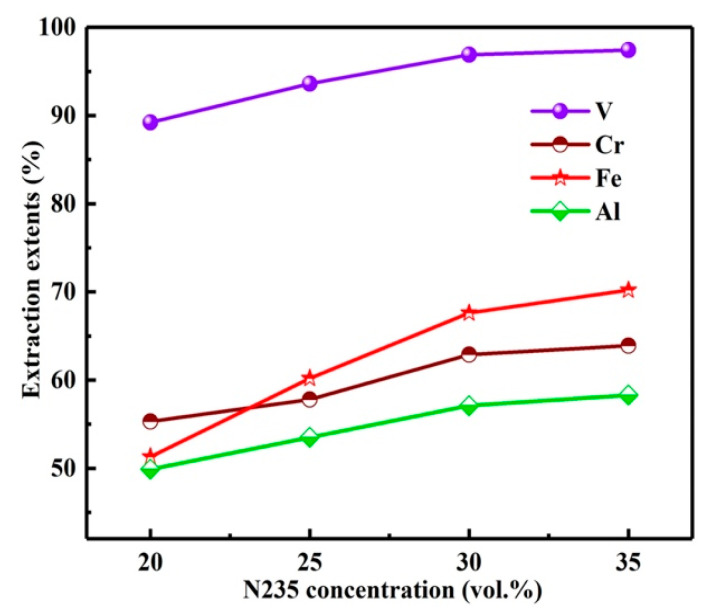
Influence of N235 extractant concentration on extraction extents.

With the O/A of 3:1, extraction time of 10 min, the influence of N235 extractant concentration on *E*s was investigated as shown in Figure 10. With the N235 concentration from 20 to 35%, the *E*s of vanadium and chromium increased from 89.2, 55.3 to 97.4, 63.9%. Meanwhile, the impurities iron and aluminum were also co-extracted with the *E*s from 51.3, 49.9 to 70.2, 58.3%. The *E* is calculated by Equation (2). However, with the increase in N235 concentration, the viscosity of organic phase was increased, which led to the slow separation of organic phase and aqueous phase. Moreover, a third phase was found in the organic phase during the extraction process. Therefore, the concentration of N235 extractant was selected as 30%.

**Figure 11:**
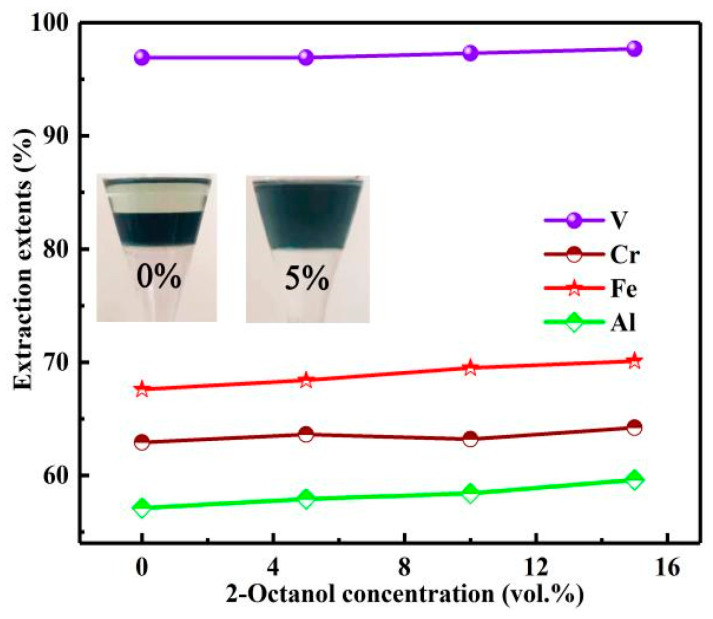
Influence of 2-octanol concentration on extraction extents.

In order to improve the performance of the organic phase, phase modifier 2-Octanol was added. The influence of 2-octanol concentration on the extraction process was studied under the conditions of N235 extractant concentration of 30%, O/A of 3:1, extraction time of 10 min. The results are shown in Figure 11.

Although the phase modifier 2-octanol did not significantly improve the *E*s of vanadium and chromium, it could evidently improve the performance of organic phase. When the addition amount of 2-Octanol was 5%, the stratification of organic phase disappeared and the phase separation rate was accelerated. Therefore, in order to further improve the phase separation rate, the 2-Octanol concentration was selected as 10%.

Based on the above research, the composition of organic phase was determined as 30% N235 + 10% 2-Octanol + 60% sulfonated kerosene. With the fixed O/A of 3:1, the influence of extraction time on *E*s was studied.

**Figure 12:**
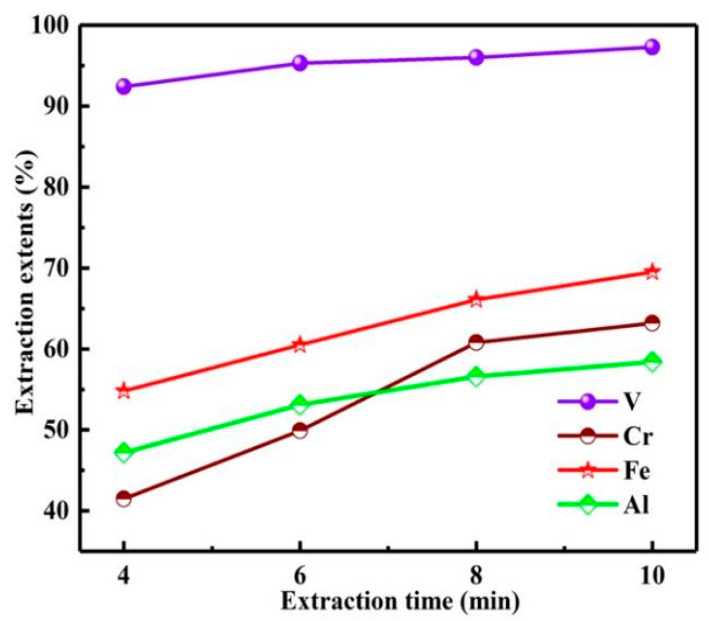
Influence of extraction time on extraction extents.

As shown in Figure 12, with the extension of extraction time from 4 to 10 min, the *E*s of vanadium and chromium gradually increased, especially the *E* of chromium. In the extraction process, with the increase in extraction time, the concentrations of vanadium ions and chromium ions in aqueous phase decreased continuously, while the concentrations of vanadium ions and chromium ions in organic phase increased gradually to equilibrium, resulting in the slow growth of *E*s after 8 min. When the extraction time was 10 min, the *E*s of vanadium and chromium were 97.3, 63.2%. Further increasing the extraction time cannot significantly improve the *E*s of vanadium and chromium, but will increase the *E*s of impurities. Therefore, the extraction time was 10 min.

The O/A is an important factor in the extraction process, which affects the *E*s and enrichment extents of vanadium and chromium. With organic phase of 30% N235 + 10% 2-octanol + 60% sulfonated kerosene, extraction time of 10 min, and the influence of O/A on the extraction extents was investigated.

**Figure 13:**
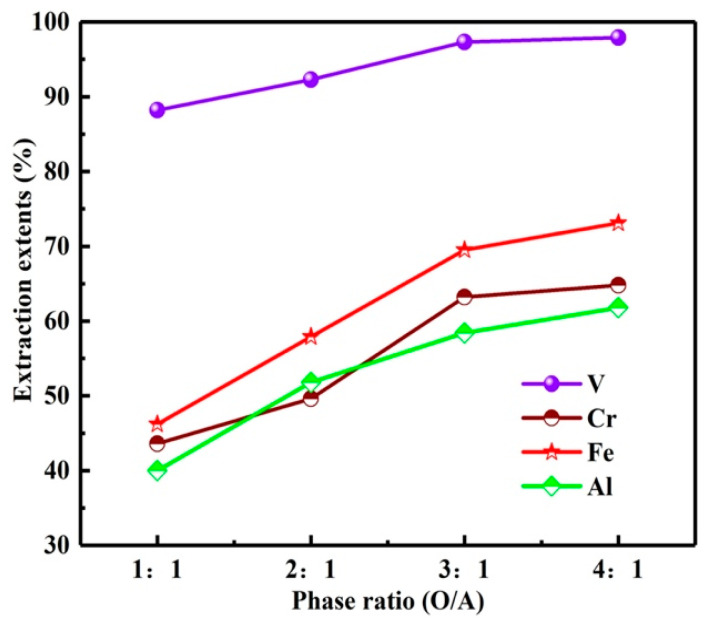
Influence of O/A on extraction extents.

As can be seen from the results in Figure 13, with the O/A increase from 1:1 to 4:1, the *E*s of vanadium and chromium increased from 88.2, 43.6 to 97.8, 64.8%. However, higher O/A is not conducive to the enrichment of vanadium and chromium. Therefore, multi-stage extraction was considered to enrich and extract vanadium and chromium at relatively low O/A. The O/A ratio was determined as 1:1 in this experiment.

#### 3.2.3. Extraction McCabe–Thiele Graph of Vanadium

**Figure 14:**
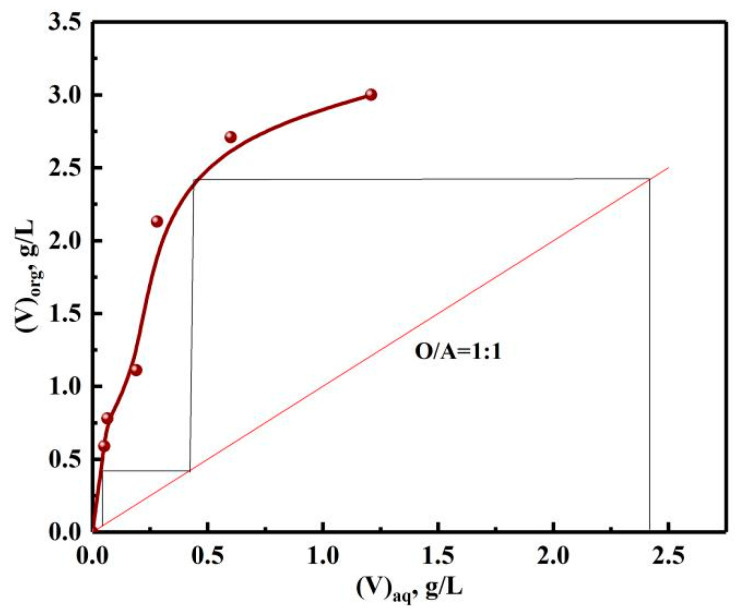
Extraction McCabe–Thiele graph of vanadium.

In order to determine the number of extraction stages, the extraction McCabe–Thiele graph of vanadium was made according to different phase ratios, as shown in Figure 14. The theoretical stages number of countercurrent extraction of vanadium was two, which was determined by the number of horizontal lines. However, in order to ensure the *E*, the actual stage was one more stage than the theoretical stages in practice. With the organic phase of 30% N235 + 10% 2-octanol + 60% sulfonated kerosene, O/A of 1:1 and extraction time of 10 min, the experiments of three-stage countercurrent extraction were performed.

As shown in Table 3, the *E*s of vanadium and chromium were 99.6 and 78.8%. Aluminum and iron were co-extracted with the *E*s of 91.2 and 95.6%, while other impurity elements were hardly extracted and remained in the raffinate. As for the impurity iron, it was further separated from vanadium and chromium in the stripping step. After extraction, vanadium and chromium were separated from a large number of impurities.

### 3.3. Stripping Vanadium and Chromium from LOP

#### 3.3.1. Mechanism Analysis of Stripping Process

**Figure 15:**
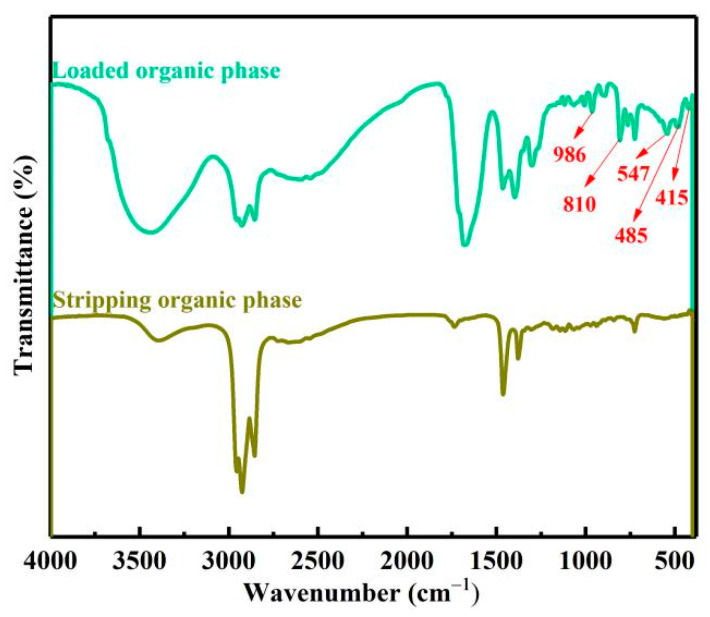
The FTIR of loaded organic phase and lean organic phase.

The stripping mechanism of vanadium, chromium, iron and aluminum ions in LOP in HCl solution was studied by FTIR, as shown in Figure 15. As discussed in the extraction mechanism, the peaks at 986, 810, 547, 485, 415 cm^−1^ belonged to V(C_2_O_4_)_3_^3−^, VO(C_2_O_4_)_2_^2−^, Cr(C_2_O_4_)_3_^3−^, Fe(C_2_O_4_)_3_^3−^ and Al(C_2_O_4_)_3_^3−^ complex ions in LOP disappeared after stripping, which proved that vanadium and chromium iron and aluminum complex ions were stripped into the stripping solution. Comparing the mechanisms of extraction and stripping, it can be seen that in the extraction process, the Cl^−^ ion in the acidified N235 extractant could be replaced by the complex anions of vanadium, chromium, iron and aluminum in the extract. In the stripping process, the complex anions of vanadium, chromium iron and aluminum loaded in the LOP could be replaced by the Cl^−^ ion in the high-concentration HCl solution. Therefore, the acidity was an important factor affecting the stripping process. However, after stripping, iron ions still existed in the organic phase judged by stripping extent. According to the relevant research [25], Fe(C_2_O_4_)_3_^3−^ in the LOP can be stripped by high concentration HCl solution, and further reacts with Cl^−^ ion to form FeCl_4_^−^ and extracted into the organic phase again. The stripping process can be expressed by Equation (10)–(16).

((R_3_NH)_3_·V(C_2_O_4_)_3_)_org_ + 3HCl = 3(R_3_NHCl)_org_ + V(C_2_O_4_)_3_^3−^ + 3H^+^ (10)

((R_3_NH)_2_·VO(C_2_O_4_)_2_)_org_ + 2HCl = 2(R_3_NHCl)_org_ + VO(C_2_O_4_)_2_^2−^ + 2H^+^ (11)

((R_3_NH)_3_·Cr(C_2_O_4_)_3_)_org_ + 3HCl = 3(R_3_NHCl)_org_ + Cr(C_2_O_4_)_3_^3−^ + 3H^+^ (12)

((R_3_NH)_3_·Fe(C_2_O_4_)_3_)_org_ + 3HCl = 3(R_3_NHCl)_org_ + Fe(C_2_O_4_)_3_^3−^ + 3H^+^ (13)

((R_3_NH)_3_·Al(C_2_O_4_)_3_)_org_ + 3HCl = 3(R_3_NHCl)_org_ + Al(C_2_O_4_)_3_^3−^ + 3H^+^ (14)

Fe(C_2_O_4_)_3_^3-^ + 4Cl^−^ = FeCl_4_^−^ + 3C_2_O_4_^2−^ (15)

(R_3_NHCl)_org_ + FeCl_4_^−^ = (R_3_NHFeCl_4_)_org_ + Cl^−^ (16)

#### 3.3.2. Influence Factors of the Stripping Process

**Figure 16:**
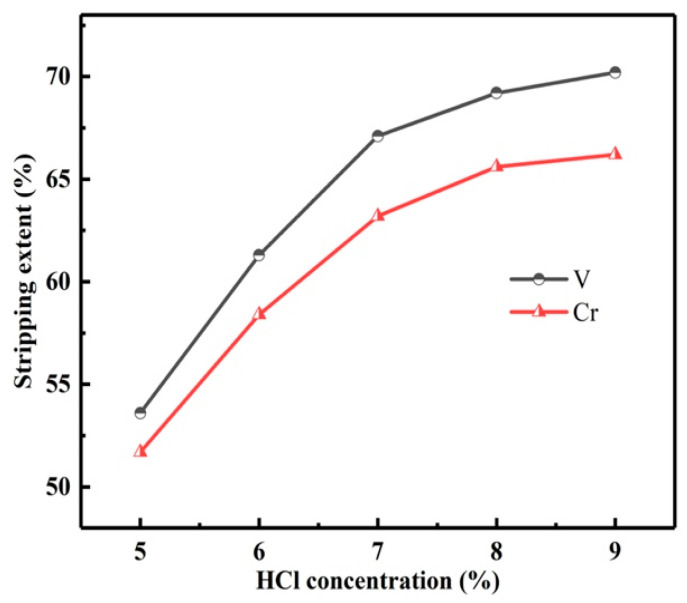
Influence of HCl concentration on stripping extents of vanadium and chromium.

In order to strip vanadium and chromium from the LOP, the stripping experiments were carried out. With the O/A of 3:1 and time of 5 min, the influence of HCl concentration on the *S*s of vanadium and chromium was studied, as shown in Figure 16.

With the HCl concentration from 5 to 7 mol/L, the *S*s of vanadium and chromium increased rapidly. However, with the HCl concentration higher than 7 mol/L, the *S*s of vanadium and chromium increased slowly. The *S* is calculated by Equation (3). Therefore, the HCl concentration was determined to be 7 mol/L.

**Figure 17:**
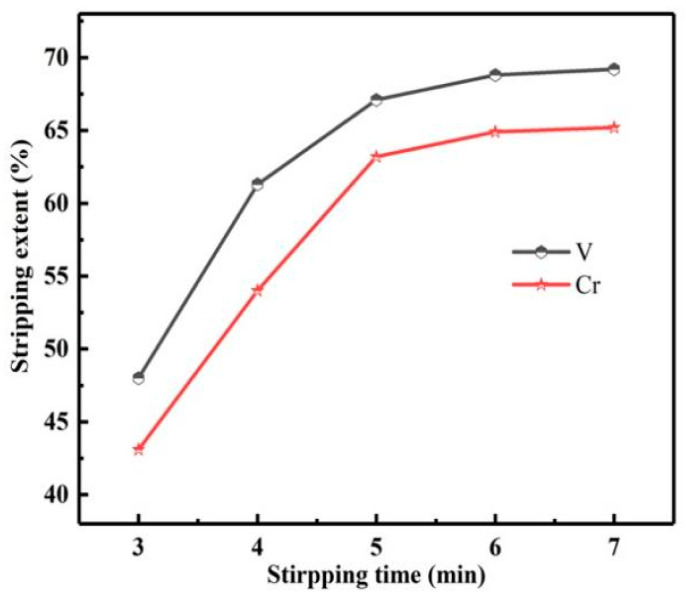
Influence of stripping time on stripping extents of vanadium and chromium.

The reaction time is another important factor affecting the *S*s of vanadium and chromium. Therefore, the influence of reaction time on the *S*s of vanadium and chromium was studied, as shown in Figure 17.

Prolonging the stripping time was conducive to the *S*s of vanadium and chromium, but the *S*s of vanadium and chromium increased slowly with the time more than 5 min. Therefore, multistage stripping could be considered to improve the *S*s of vanadium and chromium.

#### 3.3.3. Stripping McCabe–Thiele Graph of Vanadium

**Figure 18:**
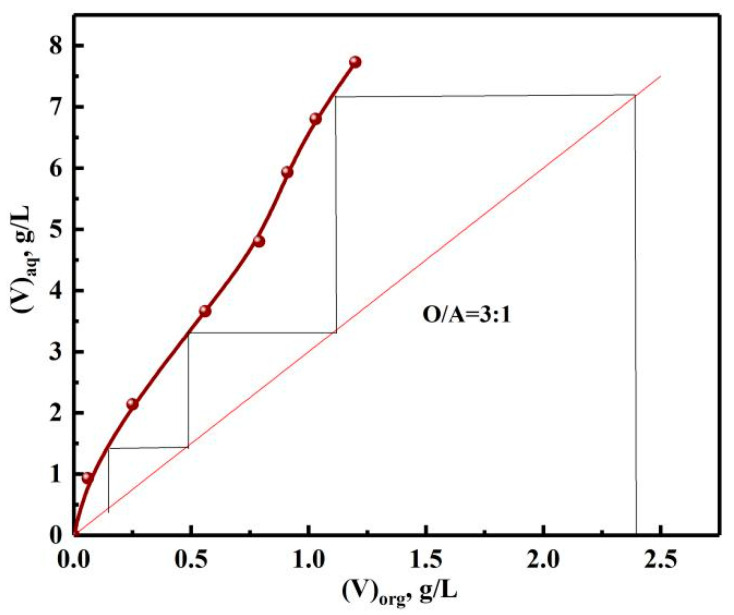
Stripping McCabe–Thiele graph of vanadium.

In order to determine the number of stripping stages, the stripping McCabe–Thiele graph of vanadium was drawn according to different phase ratios. As depicted in Figure 18, the theoretical stage number of countercurrent stripping of vanadium was three, which was determined by the number of horizontal lines. Similarly, four stages were required to ensure the *S* of vanadium.

With HCl concentration of 7 mol/L, O/A=3:1, and stripping time of 5 min, the *S*s of vanadium, chromium were 99.7 and 99.3%. The components of the stripping solution are shown in Table 4. The results show that vanadium and chromium were separated from impurities and enriched from 2.42, 1.14 to 7.15, 2.68 g/L.

### 3.4. Hydrothermal Precipitation of Vanadium and Chromium

**Figure 19:**
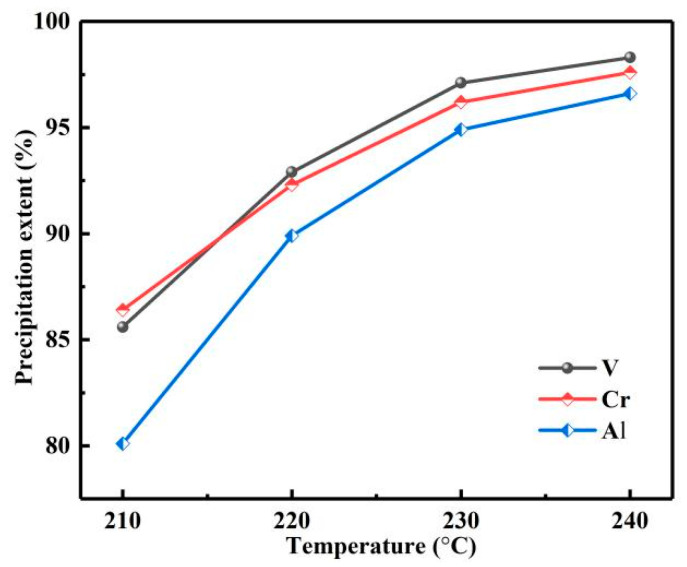
Influence of reaction temperature on precipitation extents.

**Figure 20:**
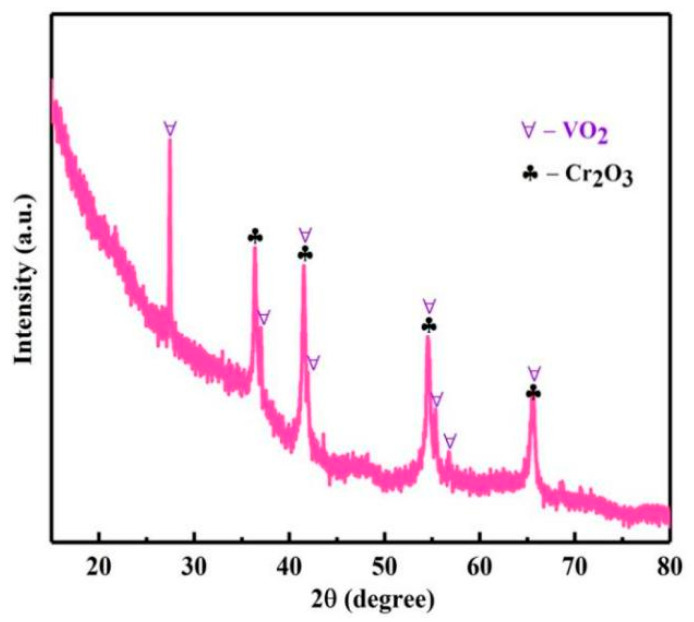
XRD analysis of the product.

With the reaction temperature from 210 to 240 °C, pH about 0.7, reaction time of 12 h, and reaction pressure of 2.9, 3.3, 3.9, and 4.6 Mpa. The influence of reaction temperature on *η*s was investigated, as shown in Figure 19. With the increase in temperature, vanadium, chromium and aluminum ions were coprecipitated. When the reaction temperature was 240 °C, the *η*s of vanadium, chromium and aluminum were 98.3, 97.6 and 96.5%, respectively. Then, the phase of the product was analyzed by XRD, as shown in Figure 20; the product was composed of VO_2_ and Cr_2_O_3._ The obtained mixture of vanadium, chromium and a small amount of aluminum is a good raw material for the production of V-Cr based alloy.

## 4. Conclusions

The process developed in this study is plausible for separating the titanium, vanadium and chromium sequentially from the oxalic acid leachate of VS. The detailed steps and results of the process are as follows:

1. The titanium in the pregnant leachate can be precipitated by a hydrothermal method. With the reaction temperature of 150 °C and reaction time of 2.5 h, more than 99% titanium was precipitated in the form of spherical anatase TiO_2_ with the purity of 95.7%.

2. With three-stage extraction of acidified N235 extractant and four-stage stripping of HCl solution, more than 99% vanadium and 78% chromium in the TFF can be transferred and enriched in the stripping solution. The concentration of vanadium was improved from 2.42 to 7.15 g/L, and chromium from 1.14 to 2.68 g/L.

3. The vanadium and chromium in the stripping solution can be coprecipitated by a hydrothermal method. Under the reaction temperature of 240 °C and reaction time of 12 h, the *η*s of vanadium, chromium and aluminum were 98.3, 97.6 and 96.5%, respectively. The product is mainly composed of VO_2_, Cr_2_O_3_ and a small amount of aluminum.

Using this process, the titanium, vanadium and chromium in oxalic acid system can be separated in the form of TiO_2_ and mixtures of VO_2_ and Cr_2_O_3_. Furthermore, the TiO_2_ can be used to prepare pigment titanium dioxide, and the mixtures of VO_2_ and Cr_2_O_3_ can be used as raw material for the production of V-Cr based alloy.

## Figures and Tables

**Table 1:** Chemical components of the leachate (g/L).

Elements	V	Ti	Cr	Fe	Mn	Si	Ca	Mg	Al
Content/g/L	2.42	2.30	1.14	0.31	0.79	0.47	0.42	0.29	0.35

**Table 2:** Chemical composition of the product (wt%).

Elements	V_2_O_5_	TiO_2_	Cr_2_O_3_	Fe_2_O_3_	MnO	SiO_2_	CaO	MgO	Al_2_O_3_
Content/wt%	2.44	95.7	0.1	0.24	0.15	1.27	0.04	0.03	<0.01

**Table 3:** Experimental results of three-stage countercurrent extraction.

Elements	Extract (mg/L)	Raffinate (mg/L)	Extraction Extent (%)
V	2396	9.5	99.6
Cr	1143	242.4	78.8
Al	356.3	31.4	91.2
Fe	270.2	11.9	95.6
Mn	786.6	738.6	6.1
Si	360.1	357.9	0.6
Ca	423.4	382.3	9.7
Mg	296.2	270.7	8.6

**Table 4:** Chemical composition of four-stage countercurrent stripping solution.

Elements	V	Cr	Al	Fe	Ca	Mg	Mn	Si
Content/g/L	7.15	2.68	0.97	0.04	0.10	0.07	0.13	0.08

## Data Availability

Not applicable.

## Abbreviations

VS	vanadium slag
TFF	titanium-free filtrate
LOP	loaded organic phase
*η*	precipitation extent (%)
*C_r_*	ion concentration in liquid before hydrothermal experiment (g/L)
*C_f_*	ion concentration in liquid after hydrothermal experiment (g/L)
*V_r_*	volume of liquid before hydrothermal experiment (L)
*V_f_*	volume of liquid after hydrothermal experiment (L)
*E*	extraction extent (%)
*C*_0_	ion concentration in extract (g/L)
*C*_1_	ion concentration in raffinate (g/L)
*S*	stripping extent (%)
*V′_Aq._*	volume of stripping solution (L)
*V_Org._*	volume of LOP (L)
*C′_Aq._*	ion concentration in stripping solution (g/L)
*C_Org._*	ion concentration in organic phase (g/L)

## References

[1] Xue N.N., Zhang Y.M., Huang J., Liu T., Wang L.Y. (2017). Separation of impurities aluminum and iron during pressure acid leaching of vanadium from stone coal. J. Clean. Prod..

[2] Fang H.X., Li H.Y., Xie B. (2012). Effective Chromium Extraction from Chromium-containing Vanadium Slag by Sodium Roasting and Water Leaching. Isij Int..

[3] Smirnov L.A., Kushnarev A.V., Fomichev M.S., Rovnushkin V.A. (2013). Oxygen-converter processing of vanadium-bearing hot metal. Steel Transl..

[4] Wen J., Jiang T., Liu Y., Xue X. (2018). Extraction Behavior of Vanadium and Chromium by Calcification Roasting-Acid Leaching from High Chromium Vanadium Slag: Optimization Using Response Surface Methodology. Miner. Process. Extr. Metall. Rev..

[5] Zhang J., Zhang W., Xue Z. (2017). Oxidation Kinetics of Vanadium Slag Roasting in the Presence of Calcium Oxide. Miner. Process. Extr. Metall. Rev..

[6] Lin X., Wang X., Cao H. (2016). High-efficient extraction of vanadium and its application in the utilization of the chromium-bearing vanadium slag. Chem. Eng. J..

[7] Wen J., Jiang T., Xu Y.Z., Liu J.Y., Xue X.X. (2018). Efficient Separation and Extraction of Vanadium and Chromium in High Chromium Vanadium Slag by Selective Two-Stage Roasting-Leaching. Met. Mater Trans B.

[8] Liu B., Du H., Wang S.N., Zhang Y., Zheng S.L., Li L.J., Chen D.H. (2013). A novel method to extract vanadium and chromium from vanadium slag using molten NaOH. AICHE J..

[9] Dong Z.H., Zhang J., Yan B.J. (2021). Co-extraction of Vanadium Titanium and Chromium from Vanadium Slag by Oxalic Acid Hydrothermal Leaching with Synergy of Fe Powder. Met. Mater Trans B.

[10] Chen X., Zhang J., Yan B. (2021). A clean method of precipitation vanadium from the vanadium bearing oxalic acid leaching solution. Miner. Eng..

[11] Kang Q., Zhang Y., Bao S. (2019). An environmentally friendly hydrothermal method of vanadium precipitation with the application of oxalic acid. Hydrometallurgy.

[12] Kumbour P., Sikong L. (2013). Effect of Oxalic Acid and Temperature on Hydrothermal VO_2_ (B) Transformation to VO_2_ (M). Adv. Mater. Res..

[13] Kim H.I., Lee K.W., Mishra D., Yi K.M., Hong J.H., Jun M.K., Park H.K. (2014). Separation and recovery of vanadium from leached solution of spent residuehydrodesulfurization (RHDS) catalyst using solvent extraction. J. Ind. Eng. Chem..

[14] Liu Z., Huang J., Zhang Y., Liu T., Luo D. (2020). Separation and recovery of vanadium and aluminum from oxalic acid leachate of shale by solvent extraction with Aliquat 336. Sep. Purif. Technol..

[15] Liu H.L., Hu J.H., Wang H., Wang C., Li J.Q. (2012). Phase transformation in hydrogen reduction of copper slag. Chin. J. Process Eng..

[16] Botto I.L., Vassallo M.B., Baran E.J., Minelli G. (1997). IR spectra of VO_2_ and V_2_O_3_. Mater. Chem. Phys..

[17] Tu S.H., Luo Z.C., Liu T., Zhu X.P., Du J. (2016). Preparation of different morphologies α-Fe_2_O_3_ by hydrothermal method. Mater. Rep..

[18] Liu S., He X., Wang Y., Wang L. (2020). Cleaner and effective extraction and separation of iron from vanadium slag by carbothermic reduction-chlorination-molten salt electrolysis. J. Clean. Prod..

[19] Liu X., Xie G., Chi H., Qian X., Zhang Y., Luo Y. (2008). A facile method for preparing VO_2_ nanobelts. Mater. Lett..

[20] Mittal J., Konno H., Inagaki M. (1998). Synthesis of graphite intercalation compounds with CrVI compounds using CrO_3_ and HCl at room temperature. Synth. Met..

[21] Hu P., Zhang Y. (2021). Mechanism of vanadium selective separation from iron in shale under an environmentally friendly oxalate ligand system. Sep. Purif. Technol..

[22] Fujita J., Martell A., Nakamoto K. (1962). Infrared Spectra of Metal Chelate Compounds. VI. A Normal Coordinate Treatment of Oxalato Metal Complexes. J. Chem. Phys..

[23] Selbin J., Holmes L.H., Mcglynn S.P. (1963). Electronic structure, spectra and magnetic properties of oxycations—IV ligation effects on the infra-red spectrum of the vanadyl ion. J. Inorg. Nucl. Chem..

[24] Kharitonov Y.Y., Buslaev Y.A. (1962). Infrared absorption spectra of oxyfluorides of some metals of the fourth and fifth groups of the periodic table. Russ. Chem. Bull..

[25] Liu Z., Huang J., Zhang Y., Liu T., Zheng Q. (2020). Separation and recovery of vanadium and iron from oxalic-acid-based shale leachate by coextraction and stepwise stripping. Sep. Purif. Technol..

